# A community-based qualitative study of perspectives on alcohol public health interventions and experiences of alcohol use

**DOI:** 10.1186/s12889-026-28513-8

**Published:** 2026-07-17

**Authors:** Sarah Dance, Andrew Weyman, Sally Adams, Amy H Barton, Amber Burd, Naomi Nelson, Charlotte Dack

**Affiliations:** 1 Department of Psychology, University of Bath, Bath, BA2 7AY UK; 2 School of Psychology, University of Birmingham, Birmingham, B15 2TT UK

**Keywords:** Alcohol, Alcohol harm paradox, Socioeconomic position, Trust, Alcohol availability, Stress, Coping

## Abstract

**Background:**

The alcohol harm paradox is where lower socioeconomic position (SEP) groups experience higher rates of alcohol-related health consequences than higher SEP populations, despite drinking less alcohol. The reasons behind this are not certain, and limited research has examined the acceptability of alcohol interventions. This study aimed to inform alcohol public health interventions targeted to lower SEP individuals by addressing two research questions: (1) What are the perspectives on alcohol public health interventions? (2) What are the experiences of alcohol use?

**Methods:**

Six focus groups (*n* = 23) were conducted in community settings with individuals who drink alcohol. We recruited from community organisations located in relatively disadvantaged areas (identified by Index of Multiple Deprivation) and workplaces with routine and manual occupations. Questions explored were the: (1) acceptability of UK alcohol public health interventions and (2) behavioural drinking patterns and reasons for drinking. Inductive and deductive framework thematic analysis was used to develop two themes.

**Results:**

Theme one highlighted a lack of trust towards the government’s motivations for implementing alcohol interventions, including perceptions that the government does not care about alcohol-related health harms and that perceived unnecessarily high alcohol-related taxes represent the reason for its legal status. Perceived authoritative interventions and interference with free will was resented. There was scepticism towards the perceived effectiveness of interventions, including perceptions of negative outcomes and that interventions do not address the underlying reasons for alcohol use. Theme two highlighted social environmental facilitators of and barriers to alcohol use, including high alcohol availability locally. Motivations for and patterns of alcohol use were outlined, including alcohol use as a coping strategy for daily life stress and preferences for cheap high strength alcohol.

**Conclusions:**

Lack of trust in government alcohol intervention was salient in this sample and may be an important consideration for intervention design. Future research could examine levels of trust in government for providing alcohol health advice and how this may be affected by SEP, and which intervention sources would be most acceptable to different SEP populations. Tailored interventions could address high alcohol availability in underserved communities, and specific motivations for and patterns of alcohol use.

**Supplementary Information:**

The online version contains supplementary material available at https://doi.org/10.1186/s12889-026-28513-8.

## Background

The health consequences of alcohol consumption are disproportionately experienced by lower socioeconomic position (SEP) populations. The alcohol harm paradox is where lower SEP populations experience higher rates of alcohol-related harms, despite drinking less alcohol than higher SEP populations [1, 2]. A systematic review of hypotheses for the alcohol harm paradox identified several explanations, including a higher rate of stressful events and coping drinking motives, a pattern of multiple health behaviours (e.g., concurrent smoking), a higher rate of heavy episodic drinking, and increased alcohol outlet density in disadvantaged areas [3]. Therefore, the higher rate of alcohol-related harm among lower SEP populations may be explained in part by psychological, behavioural, and social aspects of alcohol use. This aligns with a health psychology approach to explaining health outcomes based on the biopsychosocial model, which proposes that health cannot be explained by solely biomedical factors [4].

Social [5] and commercial [6] determinants of health may also influence the alcohol harm paradox. Within Boyd et al.’s (2022) review of explanations proposed for the alcohol harm paradox, explanations included reduced resources to counteract stressors and fewer healthcare services in disadvantaged neighbourhoods. Further identified explanations included increased alcohol availability and alcohol industry funding of misleading research. Moreover, another explanation related to a political focus on health behaviours instead of wider determinants of health which was suggested to contribute to health inequalities among disadvantaged groups [3]. Consequently, approaches to address the alcohol harm paradox may need to consider broader societal factors affecting alcohol-related health inequalities and the underlying causes of individual health behaviours related to increased health harms [7].

An emerging body of quantitative, predominantly epidemiological, literature has provided insights into the alcohol harm paradox and its potential mechanisms [1, 2, 8–11]. Studies analysing data from the Alcohol Toolkit Study (a population survey in the UK) dataset have demonstrated associations between lower SEP and increased risk of alcohol-related harm [8, 9], demonstrating the alcohol harm paradox. Analysis of the Health Survey for England (a population survey in England) indicated that whilst lower SEP individuals were less likely to drink above the recommended consumption guidelines than higher SEP individuals, they were also more likely to drink alcohol at the highest levels of consumption [10]. Another English survey indicated that lower SEP individuals had greater likelihood of reporting alcohol use, tobacco use, low exercise, poor diet, and be overweight [2]. Moreover, a systematic review found that approximately 15–30% of socioeconomic inequalities in mortality were explained by heavy episodic drinking [1]. These studies provide support for explanations of the alcohol harm paradox focused on behavioural drinking patterns and a pattern of multiple health behaviours and risks [3].

In addition, a growing body of qualitative, largely sociological, literature has generated understanding of alcohol use among different social classes [12–17]. For example, interview studies with Australian women have found that, for working class women, alcohol was perceived to relieve stress and provide comfort in the context of insecure work [12], and to reduce loneliness and promote social benefits and to cope with traumatic and stressful experiences [13]. Another interview study with Australian women identified that more affluent women had access to varied strategies to promote wellbeing (e.g., exercise), whereas working class women had less access to these strategies and used alcohol for wellbeing purposes [14]. A further interview study with individuals of lower socioeconomic status suggested that family drinking culture influences alcohol use [17]. These studies align with explanations of the alcohol harm paradox focused on coping drinking motives in response to stress and reduced protective resources for managing life events, as well as social influences [3].

The World Health Organization recommends taxes on alcohol products and bans or restrictions on alcohol advertising and availability. Other recommended interventions include minimum alcohol prices and provision of health harm information on alcohol products [18]. In the UK, the Government’s Alcohol Strategy of 2012 has informed the public health approach to alcohol. Its recommendations include implementation of: a minimum unit price (MUP) policy for alcohol, public health campaigns to provide support for individuals to change their drinking behaviour, and information about the unit content of alcohol products [19]. National guidelines on alcohol unit consumption have recommended since 2016 that all adults should not consume more than 14 units of alcohol per week [20]. However, evaluations of public awareness of these guidelines highlighted that lower SEP individuals were less likely to be aware of the guidelines following one month of implementation [21], as well as after six years of implementation [22].

MUP has been implemented in Scotland and Wales, but not in England or Northern Ireland to date. In Scotland, evaluation of MUP indicates that alcohol sales were reduced by 3% after three years [23], and deaths caused by alcohol were reduced by 13.4% after 32 months [24]. In Wales, evidence on similar outcomes are not currently available but evaluation after 42 months indicated that approximately 50% of 186 survey respondents reported no change in alcohol consumption frequency and quantity [25]. In terms of outcomes by SEP, deaths caused by alcohol reduced by 21.6% among the 10% most disadvantaged areas in Scotland following MUP [24]. However, increased economic constraints were reported in Scotland [26], as well as increased wine consumption by 1.33% points in Wales [27], among lower SEP populations. In addition, studies conducted prior to implementation identified limited public support towards MUP among the general population due to perceptions that it would be unfair [28] and not address the underlying societal and cultural influences on alcohol use [29]. This may reflect that more restrictive health interventions tend to be less acceptable [30, 31]. Whilst evaluations demonstrate positive outcomes of MUP [23, 24], there is a need to explore attitudes towards MUP among lower SEP groups to assess its acceptability.

Public health campaigns providing support for individuals to change their alcohol use have been implemented in the UK. In 2018, Public Health England and DrinkAware (an alcohol-industry funded organisation [32]) ran a campaign called Drink Free Days which promoted a reduction in the number of days on which alcohol is consumed [33]. The charity Alcohol Change UK has also run a public health campaign called Dry January since 2013, which promotes no alcohol consumption during January [34]. The campaign has been supported by the previous national health organisation Public Health England [35, 36], as well as local governments regionally [37, 38]. Evaluation of Drink Free Days found that there were no significant changes in alcohol consumption between before and after the campaign [39]. Evaluation of Dry January found a reduced AUDIT-C score [40] of at least 10% after six months among 59% of registered participants in 2019 [41]. However, evaluations of these campaigns have not examined potential differences in effectiveness between SEP populations. Moreover, participants in campaigns promoting alcohol abstinence for one month (e.g., Dry January) tend to be of higher SEP [42]. This suggests that there is a need to examine attitudes towards alcohol public health campaigns among lower SEP populations to assess acceptability.

A useful addition to the literature on the alcohol harm paradox is the use of an applied health intervention perspective to inform the design and implementation of tailored alcohol public health advice, campaigns, and policies [43, 44]. A health psychology approach based on the biopsychosocial model [4] presents a fruitful additional lens to the literature investigating alcohol use among lower SEP populations, given that this approach aims to identify targets to inform intervention design [43, 44]. However, research examining the alcohol harm paradox has not explicitly applied a health psychology lens to date, to our knowledge. Moreover, there is limited research examining the acceptability of alcohol interventions in the UK among lower SEP populations. Health intervention development frameworks highlight a need to explore the experiences of target populations to design tailored interventions [43, 44]. The person-based approach to developing interventions recommends that their design should be informed by qualitative research with a target population to identify requirements of the target population and to explore their attitudes towards interventions. This enables interventions to be based on a target population’s experiences with a health behaviour (e.g., alcohol use) and their attitudes towards interventions (e.g., the UK-based alcohol public health interventions outlined above) [44].

However, research tends to recruit lower SEP populations less effectively [45], which indicates that typical recruitment strategies may be less effective among underserved populations. Barriers to research recruitment may include less accessible research locations, delayed reimbursement, previous negative contact with universities, and experience of unethical research practices [46]. Other barriers may include inaccessible research materials, a lack of researcher time or expertise in effective recruitment for disadvantaged populations, and reduced trust towards researchers and the research itself. Further barriers may relate to issues with transportation and time constraints preventing attendance at typical research locations and times [47]. Therefore, alternative approaches are likely needed to effectively recruit underserved populations to alcohol-related health research.

Several strategies have been suggested to improve the recruitment of underserved populations to research. Guidance for involving underserved community groups in health research emphasises a need to visit groups in person to develop relationships and trust, and to carry out research at a groups regular location and time [48]. Evidence suggests that inclusive recruitment strategies should recruit via community gatekeepers [49] and conduct research in familiar locations to participants [50]. Developing connections with communities [46] and increasing familiarisation of researchers to potential participants [47] is suggested to enhance trust towards research participation among disadvantaged populations.

This study was informed by the person-based approach to intervention development [44], with a view to inform the development of alcohol public health interventions targeted to lower SEP populations. We used a community-based qualitative approach to explore perspectives on alcohol public health interventions and experiences of alcohol use. Therefore, the research questions were:

1. What are the perspectives on alcohol public health interventions?
2. What are the experiences of alcohol use?

## Methods

### Design

This qualitative study used semi-structured focus groups, which were considered appropriate to explore and enable group discussion of perspectives and experiences around alcohol use and interventions. We reported this study using the Standards for Reporting Qualitative Research checklist [51] (see Supplementary Materials). Ethical approval was obtained from the University of Bath Biomedical Science Ethics Committee (code: 0744–3661) and all participants provided informed consent.

### Conceptualisation of socioeconomic disadvantage

This study primarily considered lower SEP via neighbourhood level disadvantage. This was based on the context of alcohol-related harm in the UK. Government statistics on alcohol-related deaths and morbidity are reported by neighbourhood level disadvantage [52] via the Index of Multiple Deprivation (IMD). This measure combines indicators of socioeconomic disadvantage, including: income, education, employment, poor health, crime, accessibility of housing and local services, and local environment quality [53]. This perspective may align with the implementation of interventions targeted towards disadvantaged neighbourhoods which experience high levels of alcohol-related harm. Furthermore, neighbourhood-level disadvantage considers socioeconomic disadvantage more broadly than an individual-level indicator (e.g., income) might by considering the context of an individual’s environment. UK-based guidance on recruiting individuals experiencing socioeconomic disadvantage to research suggests that experiences are not limited to one indicator, but that multiple social and economic components should be considered [54]. This perspective formed the basis of our recruitment strategy which targeted community organisations located in relatively disadvantaged areas according to the IMD. We were also informed by a pragmatic epistemological lens focused on which research approaches would enable us to address our research questions [55, 56]. This lens also informed our collection of additional sociodemographic data (e.g., education, income, occupation). This was because lower SEP is a multi-faceted concept and several SEP indicators exist, such as income, education, occupation, housing status, social class, wealth, and area-level measures of deprivation [57, 58]. Guidelines on measuring SEP emphasise that there is no universally accepted single measure of SEP, given that each indicator has a different theoretical basis. Moreover, SEP indicators tend to correlate, hindering the argument that a single indicator is optimal [59].

### Participants and recruitment

**Fig. 1:**
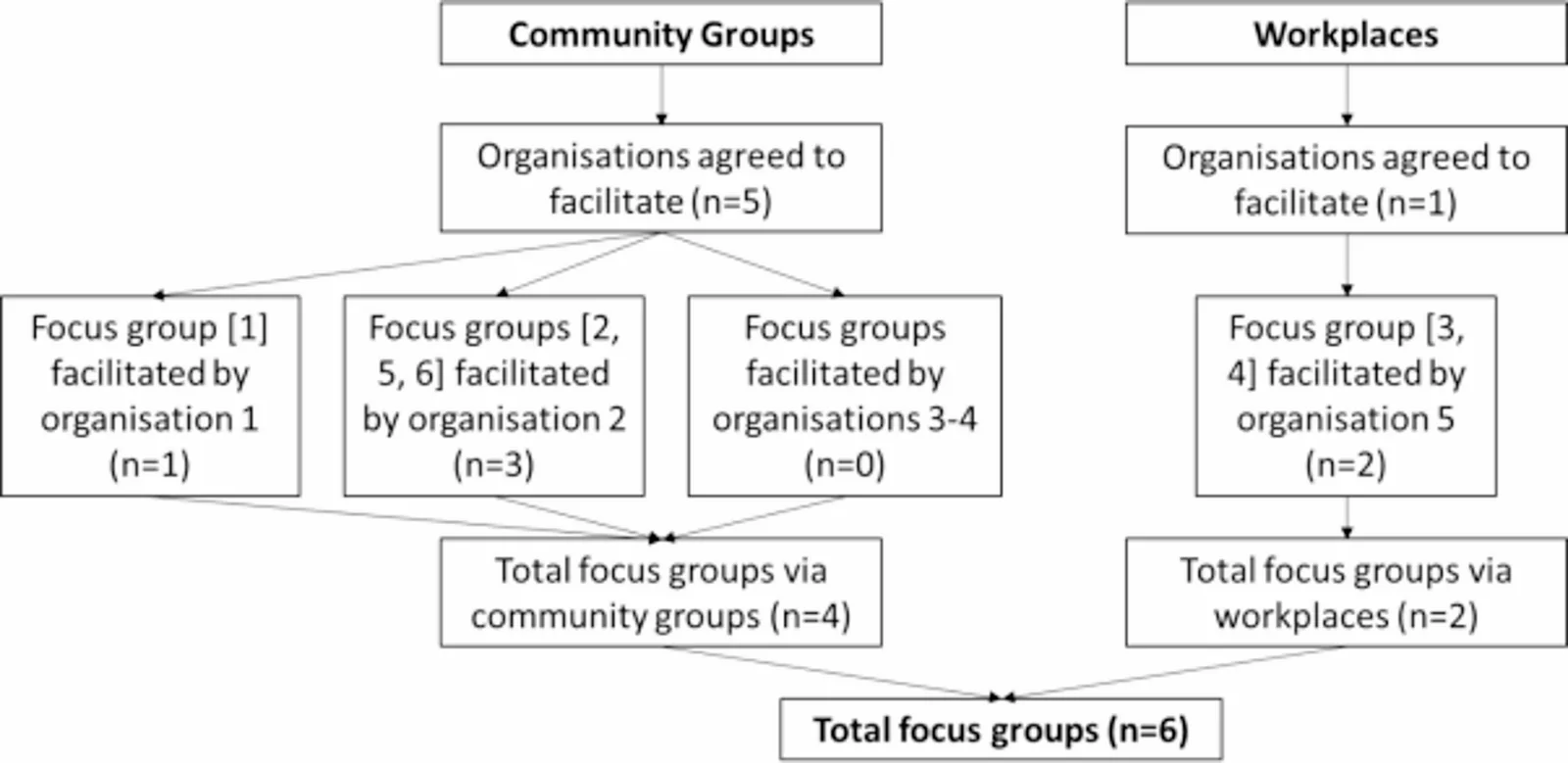
Recruitment process. For the focus groups facilitated via community organisations (*n* = 4), either: the organisation leader provided the location and time of a potential focus group, and individuals were able to attend if interested (Focus Group 1), individuals contacted the researcher to arrange a time (Focus Groups 2 and 6), or a focus group was conducted spontaneously (Focus Group 5). For the focus groups facilitated via workplaces (*n* = 2), the manager provided the location and time of a potential focus group, and individuals were able to attend if interested (Focus Groups 3 and 4)

Participants were adults aged over 18 years old who drink alcohol. We aimed to predominantly recruit individuals of lower SEP. We targeted recruitment towards community organisations located in relatively disadvantaged areas, which were identified as areas among the most disadvantaged 10% and 20% of small areas in England according to the Index of Multiple Deprivation (IMD) [53]. These community organisations (e.g., community social groups) advertised themselves as serving the local communities within their disadvantaged areas (e.g., by providing community and social space). We also targeted workplaces employing individuals mainly in routine and manual occupations [60]. Recruitment was informed by evidence on effective strategies to recruit disadvantaged populations to health research. The first component was informed by guidance on inclusive recruitment to health research via community groups (the CHecklist for Inclusive COmmunity involvement in health research (CHICO)) [48]. The second component was informed by evidence on using workplaces as a recruitment strategy for lower SEP individuals [61]. SD visited each organisation in person to informally share more information about the study, answer any questions, and ask how best to accessibly run the study with their organisation. With agreement from the organisation, the researchers then returned to invite individuals to participate and conduct focus groups (see Fig. 1).

### Data collection

Six focus groups were conducted between June and August 2024. They were audio-recorded and lasted for approximately 48 min (range 38–66 min). The first focus group piloted the topic guide (which remained the same afterwards). Each focus group was facilitated by SD. Four focus groups were supported by a second researcher (AHB, AB, NN), who made written notes on key discussion points and non-verbal communication. Reflexive notes on key areas of discussion within focus groups and group characteristics were made by the second researcher during focus groups or SD after focus groups to inform initial ideas for analysis. A paper study materials approach was adopted to aid accessibility [62, 63]. Participants were provided with a paper information sheet and gave written informed consent. Prior to participating in a focus group and after completing a consent form, participants were asked record their age, sex, gender, ethnicity, postcode (used to calculate IMD; [53]), job title (categorised using the three classes of the National Statistics Socio-economic Classification: managerial, administrative, and professional occupations; intermediate occupations; and routine and manual occupations; [60]), highest education level (categorised using UK national education levels; [64]), household income, and alcohol use (AUDIT-C questionnaire; see Supplementary Materials [40]), via a survey. We were unable to classify some of these data (IMD of four participants, education of six participants, household income of eight participants) due to limited or no information provided, as participants could freely choose whether to complete questions on a paper survey. However, we considered all participants to meet our primary objective of being recruited through a community organisation located within a relatively disadvantaged area or a workplace of routine and manual occupations. These additional data collected via the short survey were intended to provide further contextual data rather than to be used as a screening tool.

The topic guide was informed by literature on the alcohol harm paradox, alcohol public health interventions, and factors associated with alcohol use (see Table 1 for an outline and Supplementary Materials for more details). The first part focused on the acceptability of a selection of high-profile contemporary alcohol public health interventions, informed by the UK public health approach to alcohol based on the Government’s Alcohol Strategy of 2012 [19] (e.g., unit guidelines, MUP, Dry January, and Drink Free Days), to address the first research question. Participants were shown paper images of interventions to prompt discussion [65–69] (copies of images are available on reasonable request). Not all interventions were presented to every focus group, on the basis of decisions during focus groups about what was most beneficial for promoting group discussion and ensuring the duration of focus groups stayed on time. The topic guide provided a decision to provide a prompt about either the Dry January campaign or the Drink Free Days campaign. In groups where discussion was flowing, only one of these interventions was presented to allow participants to continue leading the discussion and to avoid breaking up discussion. In groups where discussion had come to a natural end about an intervention, another of these interventions was presented to prompt further discussion. Focus groups two, four, and five were presented with both Dry January and Drink Free Days. Focus group one was presented with Dry January. Focus groups three and six were presented with Drink Free Days (although Dry January was briefly raised without specific prompt by participants in focus group three). Whilst this meant that groups sometimes provided views on different interventions across the sample, we considered this approach to enable us to answer the question of overall perspectives on alcohol public health interventions. The second part of the topic guide focused on participants’ accounts of drinking patterns and reasons for drinking to address the second research question. Participants received £25 cash reimbursement, as recommended by the National Institute for Health and Care Research for public contributor involvement in research [70].

**Table 1:** Outline of areas of focus within topic guide

Topic Guide Section	Areas of focus
Part 1: Alcohol interventions	Alcohol units
	Minimum unit pricing
	Dry January
	Drink Free Days
	Licensing hours
Part 2: General introduction to alcohol consumption	The last time of alcohol consumption
Part 3: Alcohol consumption	Reasons for alcohol consumption
	Positives and negatives of alcohol consumption
	Location of consumption
	Daily life and alcohol consumption
	Price and alcohol consumption

### Sample size

Before collecting data, we estimated that approximately 20 to 25 participants may provide appropriate depth of insight to the research questions. This provisional sample size was informed by considerations that the study was relatively exploratory and the focus group design would require a moderate sample size given that each focus group would consist of several individuals. This initial sample size was also informed by a prior study addressing similar aims [71]. Whilst conducting focus groups and data analysis, we determined that 23 participants across six focus groups enabled us to address the research questions and to conduct framework thematic analysis [72] on the basis of the following considerations [73]: (i) the study was relatively exploratory; (ii) the data were rich and complex, allowing for in-depth interpretative analyses, (iii) the conduct of several focus groups provided good insights into each group; and (iv) the data were of good quality and indicated good rapport between researcher and participants, and between participants within focus groups. Ultimately, the data collected from 23 participants across six focus groups enabled an effective framework thematic analysis consisting of both interpretative and descriptive analyses, resulting in rich insights within a sufficient number of themes and depth of analysis [72].

### Sample characteristics

There were 23 participants in total across six focus groups (see Table 2). The average age was 42 years (27 to 70 range), almost three quarters were male, and the majority were British (see Table 2 for a summary of sociodemographic data). We retained the data of two participants who had AUDIT-C scores of zero as their focus group data discussed their perspectives on alcohol public health interventions and alcohol use, which allowed us to address the research questions. Moreover, the measure was used to provide contextual data rather than as a screening tool. During focus groups, both of these participants said that they “used to drink” alcohol, which indicated that they were former consumers of alcohol and supported our decision to retain their data.

**Table 2:** Sample characteristics

Group	Participant	Age	Sex	IMD	Occupational classification	Highest education level	Approximate annual household income average	AUDIT-C risk score
Focus Group 1 (*n* = 5)	FG1 P1	27	Male	10	Managerial, administrative, and professional occupations	Degree or equivalent	£80,000	3
	FG1 P2	46	Male	10	Managerial, administrative, and professional occupations	Degree or equivalent	£100,000	4
	FG1 P3	33	Male	9	Managerial, administrative, and professional occupations	A Level or equivalent	-	4
	FG1 P4	33	Male	-	Intermediate occupations	-	£40,000	3
	FG1 P5	35	Male	10	Routine and manual occupations	A Level or equivalent	-	8
Focus Group 2 (*n* = 3)	FG2 P1	57	Female	2	Unemployed	GCSE or equivalent	£15,000	5
	FG2 P2	53	Female	3	Unemployed	None	£10,000	9
	FG2 P3	47	Female	2	Unemployed	GCSE or equivalent	£10,000	12
Focus Group 3 (*n* = 4)	FG3 P1	43	Male	-	Routine and manual occupations	-	-	2
	FG3 P2	59	Male	6	Routine and manual occupations	A Level or equivalent	£25,000	6
	FG3 P3	39	Female	-	Managerial, administrative, and professional occupations	A Level or equivalent	£65,000	3
	FG3 P4	35	Female	10	Routine and manual occupations	GCSE or equivalent	-	2
Focus Group 4 (*n* = 5)	FG4 P1	45	Male	2	Routine and manual occupations	GCSE or equivalent	£40,000	6
	FG4 P2	34	Male	8	Routine and manual occupations	GCSE or equivalent	-	11
	FG4 P3	46	Male	9	Routine and manual occupations	-	£65,000	3
	FG4 P4	39	Male	10	Routine and manual occupations	A Level or equivalent	£18,000	6
	FG4 P5	35	Male	8	Routine and manual occupations	GCSE or equivalent	-	10
Focus Group 5 (*n* = 4)	FG5 P1	36	Female	2	Routine and manual occupations	-	-	0
	FG5 P2	70	Male	10	Unemployed	A Level or equivalent	-	0
	FG5 P3	55	Male	3	Unemployed	GCSE or equivalent	£6,500	3
	FG5 P4	35	Male	-	Unemployed	A Level or equivalent	£12,000	2
Focus Group 6 (*n* = 2)	FG6 P1	34	Male	6	Routine and manual occupations	-	£30,000	12
	FG6 P2	35	Male	9	Unemployed	-	£24,000	10
Summary of participant demographics across focus groups		25–34 (*n* = 5)	Female (*n* = 6)	1–2 (*n* = 4)	Unemployed (*n* = 7)	None (*n* = 1)	£0-£10,000 (*n* = 3)	0–4 (*n* = 12)
		35–49 (*n* = 13)	Male (*n* = 17)	3–4 (*n* = 2)	Routine and manual occupations (*n* = 11)	GCSE or equivalent (*n* = 7)	£10,001-£20,000 (*n* = 3)	5–7 (*n* = 4)
		50–64 (*n* = 4)		5–6 (*n* = 2)	Intermediate occupations (*n* = 1)	A Level or equivalent (*n* = 7)	£20,001-£30,000 (*n* = 3)	8–10 (*n* = 4)
		65–74 (*n* = 1)		7–8 (*n* = 2)	Managerial, administrative, and professional occupations (*n* = 4)	Degree or equivalent (*n* = 2)	£30,001-£40,000 (*n* = 2)	11–12 (*n* = 3)
				9–10 (*n* = 9)		Not able to classify (*n* = 6)	£40,001+ (*n* = 4)	
				Not able to classify (*n* = 4)			Not able to classify (*n* = 8)	

*IMD* index of multiple deprivation (a measure in the UK indicating area-level deprivation; 1 refers to more deprived and 10 refers to less deprived [53])

- = not able to classify due to limited or no information provided. Age groups are based on the Office for National Statistics categories [74]. Note that three participants (FG5 P1, FG5 P2, FG5 P4) did not complete one question each from the AUDIT-C scale (see Supplementary Materials) so their AUDIT-C scores are based on available responses

### Data analysis

Framework thematic analysis was used, informed by Gale et al.’s (2013) guidance [72]. This method is suitable for applied health research [75], with advantages derived from its ability to generate understanding of a phenomenon and to identify ways to improve interventions [72]; consistent with the person-based approach to intervention development [44]. Framework thematic analysis is a type of thematic analysis which enables qualitative codebook analysis to be conducted. It allows for both interpretative and descriptive analyses [72, 75, 76]. We therefore considered it appropriate for our topic guide and data collection which included both the meaning of perspectives on interventions (theme one) and accounts of alcohol use which were closer to the surface of the data (theme two). Moreover, the method is not situated within one theoretical or epistemological perspective. This flexibility enabled us to conduct both an inductive (codes arose from the data collected, e.g., mainly in theme one) and deductive (codes based on existing literature, e.g., mainly in theme two) analysis [72]. Our analysis was informed by a critical realist perspective, which combined the viewpoints that an observable reality exists (ontological realism), but that this reality is shaped by individual experiences and social structures (epistemological relativism) [77]. Given that this study was guided by the person-based approach to intervention development, our analysis focused on identifying factors (in particular, psychological, social, and behavioural factors [4]) which could represent targets for interventions [44].

Following the steps on conducting framework thematic analysis outlined by Gale et al. (2013) [72], audio recordings were transcribed and anonymised. During the process of data collection, the researchers conducting data collection discussed and noted key topics discussed by each focus group to inform initial thinking for analysis. Transcripts were inductively coded by SD and AHB independently, who met to compare codes. Given that SD and AHB had sometimes used codes with different names for the same idea, the meeting for comparison of coding included finalisation of agreed code names. Deductive codes were also developed from the existing literature by SD. This inductive and deductive coding informed the development of a draft analytical framework. The framework was reviewed by CD and at this stage some codes were amended (e.g., a few codes were combined where they were considered to capture the same concept). The framework was then piloted by SD and AHB on two transcripts. Following this pilot, some codes were amended (e.g., a few codes were again combined if they were seen to capture the same concept and a few codes were added if they were seen to capture concepts not already captured by the framework). An audit trail of the coding process and framework development was noted. Following this agreement on final codes, we considered the analytical framework to be appropriate to code the dataset. Next, SD applied the analytical framework to all transcripts using NVivo. A framework matrix of coded data was generated by NVivo. The matrix facilitated interpretations and discussions of data, which led to the development of two themes which aimed to identify common meaning across focus groups: (1) Lack of trust in government alcohol intervention, and (2) Psychosocial context of alcohol use. The development of themes was fully conducted by the research team and member reflection was not used in this study due to a relationship with participants not existing beyond data collection. This approach was taken to reduce participant burden during recruitment. Themes were developed by SD and presented to the research team for discussion in meetings. These included meetings between (i) the data collection team (SD, AHB, AB, NN) and the main supervisor (CD) and (ii) SD and the supervisors (CD, AW, SA). The overall themes stayed the same after these meetings but the use of discussion aimed to add further reflection to the analysis.

### Researcher positioning and reflexivity

Epistemological relativism, as part of our critical realist perspective [77], suggests that our interpretation of the data is influenced by our own experiences. SD, who facilitated the focus groups and led data analysis, was a PhD student with a background in health psychology and training in qualitative research methods. CD has a background in health psychology and health services mixed-methods research. AW has a background in the psychology of risk and behaviour change mixed-methods research. SA has a background in health and experimental psychology research. AHB and AB also have a background in health psychology, and NN in psychology. These research backgrounds may have influenced the focus on psychosocial aspects of alcohol use. We acknowledge that our analysis has developed from a mainly outsider position, as the authors would be categorised as middle SEP using current indicators such as education and occupation. We considered how our experiences of alcohol use and socioeconomic backgrounds may have influenced how we interpret the data.

## Findings

Two themes were developed (see Table 3). Theme one addressed the first research question and theme two addressed the second research question.

**Table 3:** Summary of themes and sub-themes

Themes	Sub-themes	Summary
Lack of trust in government alcohol intervention	Lack of trust towards motivations of government alcohol intervention	This sub-theme refers to a lack of trust towards why the government intervenes on alcohol use. It includes perceptions that alcohol’s legal status is driven by the financial gains from alcohol taxes, and that the government does not care about the health consequences of alcohol use.
	Scepticism about the effectiveness of government alcohol intervention	This sub-theme refers to sceptical attitudes that interventions would be effective. Perceptions of negative outcomes of interventions, and some misunderstanding of intervention objectives, led to sceptical attitudes. It includes some recommendations for developing interventions which were seen to be effective.
Psychosocial context of alcohol use	Social environmental facilitators of and barriers to alcohol use	This sub-theme refers to societal factors which were seen to promote or hinder alcohol use, including alcohol availability, social norms, family, work, and the COVID-19 pandemic.
	Motivations for and patterns of alcohol use	This sub-theme refers to underlying motivations for alcohol use, and behavioural drinking patterns of alcohol use. It includes coping drinking motives for managing stress in daily life. It also includes patterns of heavy episodic drinking and preferences for cheap high strength alcohol.

### Theme 1: Lack of trust in government alcohol intervention

There was an overarching lack of trust towards government alcohol intervention. This theme encompasses lack of trust towards the motivations for interventions and scepticism about their perceived effectiveness.

#### Sub-theme 1a: Lack of trust towards motivations of government alcohol intervention

Overall, participants expressed doubt that the government had good intentions for implementing interventions on alcohol use, leading to a lack of trust towards the presented interventions. Some participants suggested that there were unnecessarily high taxes on alcohol, as well as tobacco. Some participants perceived the government to be taking money away from people already struggling financially. They reported that they were aware of the health and social harms associated with alcohol use, and saw tax gained as a key reason for its legal status and tax-related government intervention despite these harms.

*“It [alcohol] isn’t*,* expensive to make*,* it’s just more of that money is tax. So the government steep more money out of people who are tryna get by. I mean they tax it enough as it is.” (Focus Group 3; FG*,* Participant 2)*.

A few participants suggested that the government does not care and *“aren’t bothered” (FG5*,* Participant 3)* about the health consequences of alcohol sales, and that the government cared more about the financial profits from alcohol sales. One participant suggested that the health impacts of alcohol are beneficial for the government due to a depopulation effect, and perceived that healthcare professionals benefitted financially from treating patients with alcohol-related harm.

*“It’s like*,* if people didn’t get sick [from alcohol]*,* do you know what I mean. The doctors*,* they’d be making no money*,* it’s all money money money.” (FG5*,* Participant 1)*.

A few participants compared alcohol to other drugs and their associated harms. Cannabis was suggested to be associated with less harms due to effects of increased relaxation and reduced anti-social behaviour, and so the government was suggested to not be able to financially profit from cannabis harms. One participant suggested that alcohol and tobacco had legal status due to their ability to generate revenue for the government despite death rates.

*“Heroin*,* cocaine*,* all them drugs are banned*,* they’re illegal ‘cause they kill people. Alcohol and cigarettes kill people but they’re not banned*,* ‘cause the government can make money off of the tax. And they’d rather use your tax money to tell you not to vape*,* even though they’re selling it to you. It’s just a big circle.” (FG5*,* Participant 3)*.

Conversely, some participants suggested that alcohol’s legal status indicated that the health harms must be limited, and some suggested that the government would make alcohol illegal if it were committed to reducing alcohol-related harms.

*“If they [the government] were really serious about people’s health and drinking problem they wouldn’t sell alcohol.” (FG3*,* Participant 2)*.

Some participants stated that government intervention for alcohol use was interfering with their free will, and they expressed resentment towards authoritative action which did not involve perceived free choice. One participant made a comparison to high alcohol use by politicians, reflecting some awareness of the alcohol harm paradox [2] (“*the politicians at night*,* they sunk more*,* ten times more than we do*”; *FG5*,* Participant 3*). They emphasised that their own alcohol use was outside the remit of where the government should intervene.

*“I could do it [Dry January]*,* but I wouldn’t want to do it ‘cause I feel like you’re being told what to do.” (FG2*,* Participant 1)*.

A few participants suggested that the money gained from alcohol tax should be put towards healthcare services (e.g., treatment services, National Health Service; NHS) rather than other expenditure. They suggested that this focus would be more useful in supporting individuals with their alcohol use than tax-related interventions.

*“More money should be put into things like [treatment services] … more money needs to be invested in places that help people*,* rather than just getting some cheap tax*,* just some free money*,* cause it’s not helping people.” (FG2*,* Participant 3)*.

#### Sub-theme 1b: Scepticism about the effectiveness of government alcohol intervention

Participants also expressed doubt that the alcohol interventions implemented by the government would be effective at reducing alcohol use. There were sceptical attitudes towards the perceived effectiveness of the presented interventions, leading to a lack of trust as participants tended to focus on perceived negative consequences of interventions and so perceive fewer beneficial outcomes. Many participants were sceptical about the perceived outcomes and effectiveness of alcohol interventions. In particular, MUP was suggested to lead people towards crime and theft. It was seen to promote crime in order to obtain money to buy alcohol, *“maybe they’re thinking if we haven’t got the money*,* and you know what happens we’ll go and rob don’t we to get the money” (FG2*,* Participant 2)*; or theft of alcohol from shops; *“the shop lifting would go up” (FG4*,* Participant 1)*. A few participants suggested that it would unfairly affect those drinking alcohol at low levels, for example, at a pub. This was seen to also have negative economic impacts on pubs and may lead to pub closures. Conversely, another participant suggested that MUP would have positive economic impacts, but were sceptical about potential benefits for the NHS.

*“I feel like that [MUP] just stops a regular person going out and spending money in a pub or something*,* probably puts more pressure on pubs and stuff to stay open because people don’t want to go out as much.” (FG1)*.

*“So I mean*,* it’s [MUP] probably driving the economy in Scotland*,* it’s probably putting money in the … economy*,* it’s not doing a lot for the NHS [National Health Service]*,* because the NHS is sinking regardless of this anyway. I don’t think it’s a deterrent … people will moan about it but they’ll still go and buy it.” (FG3*,* Participant 2)*.

Some participants suggested that the cost of alcohol was not a significant factor for individuals with high alcohol consumption, as it was suggested that they would obtain alcohol via other means. One participant reported prioritisation of alcohol above other financial outgoings.

*“All my alcohol will be prioritised over food*,* over bills*,* so I don’t think it [MUP] would make any difference … People will do anything to get their fix*,* whatever their drug choice is*,* and that would not make a difference.” (FG2*,* Participant 3)*.

A few participants suggested that rather than economic approaches such as MUP, interventions should target the underlying issues related to alcohol consumption (e.g., daily life stress). Moreover, MUP was suggested to lead individuals to *“find other cheaper ways to get off your face” (FG1)*, such as using other drugs.

A perception of MUP by many participants as a form of tax and misunderstanding of its objectives contributed to a lack of trust towards the policy and its effectiveness.

*“It’s [MUP] like the sugar tax.” (FG5*,* Participant 1)*.

One participant suggested that MUP would lead people to consume stronger alcoholic drinks with higher units, such as strong cider; *“I would think probably*,* most people who drank*,* had a drink problem*,* would probably revert to drinking strong cider at £3.25 than they would to drink*,* I dunno red wine or lager.” (FG3*,* Participant 2)*. Alternatively, some participants suggested that higher prices for alcohol would stop them from buying alcohol, with comparisons made to tobacco prices. One participant highlighted how they no longer smoked due to tobacco prices: *“It’s like smoking*,* I don’t smoke ‘cause the prices gone up*,* like it’s just ridiculous” (FG5*,* Participant 1)*. Some participants suggested that they would be unable to buy alcohol once the price was too high, and that ultimately price would be a deciding factor in purchasing alcohol.

*“It would get to the point where*,* like I ain’t paying that price … I paid like 7 and half quid for a pint before*,* not realising*,* already ordered it and was like fucking hell mate it’s not that posh in here … it is down to money.” (FG6*,* Participant 1)*.

Most participants perceived alcohol units as hard to understand, which contributed towards disinterest towards them and ultimately led to a sense of low trust. Most participants reported a lack of use and awareness of alcohol unit consumption guidelines. Units were suggested to be particularly ineffective when buying or drinking alcohol; *“It’s not a guideline that registers with anybody who’s actually physically buying drink or helping themselves to drinks at a party” (FG3*,* Participant 2)*. Some suggested that the recommended weekly maximum unit intake was lower than average consumption. One queried whether transfer of recommended units across weeks was possible, demonstrating misunderstanding of their function.

*“I’m sure most people drink more than that [group laughs].” (FG4*,* Participant 1)*.

*“So is it like if you didn’t use your 14 per week*,* you carry them over to another?” (FG2*,* Participant 1)*.

In contrast, some participants recommended that unit consumption guidelines should reference a maximum number of drinks rather than number of units. This was suggested to be easier to understand and implement when drinking alcohol.

*“I think it’d be better in glasses rather than units.” (FG4*,* Participant 1)*.

A few participants saw a recycling image [69] on the Drink Free Days campaign as an unrealistic portrayal of alcohol use, which contributed towards unconvinced and incredulous attitudes about its perceived effectiveness and a sense of lack of trust. The recycling box image was suggested to be an inaccurate depiction of occasions when high quantities of alcohol were consumed, as it was suggested that some individuals would put their alcohol products in other bins (e.g., household waste rather than recycling) to conceal it. The negative attitudes of others (e.g., neighbours, waste collectors) seeing the recycling bin was highlighted as a key reason for this concealment.

*“That image is absolutely not useful because if you’re drinking too much you don’t put all your stuff in the recycling bin.” (FG2*,* Participant 3)*.

In contrast to the sceptical attitudes towards the presented interventions, some recommendations were made for improving the perceived effectiveness of interventions. Some participants suggested that campaigns should be designed by people who drink alcohol and focus on experiences of alcohol use and its consequences; “*I think they probably need people to start the campaigns who have experienced problems with alcohol” (FG2*,* Participant 3).* Some participants recommended that there should be increased education around alcohol use (e.g., in schools) and unit consumption guidelines to raise awareness of the health consequences; “*They just gotta keep advertising that it’s bad for you” (FG5*,* Participant 2).* Some participants recommended the use of visual components in interventions, such as images depicting alcohol harms (e.g., a damaged liver) on alcohol products. Comparisons were made to the visibility of pictorial warnings on tobacco packaging, which were suggested to be effective in raising awareness of tobacco harms; “*Maybe show pictures on the bottles like they do with cigarettes” (FG4*,* Participant 3).*

### Theme 2: Psychosocial context of alcohol use

This theme contains participants’ accounts of the context of their alcohol consumption, related to social environmental factors acting as facilitators or barriers to alcohol use, and individual motivations for and drinking patterns of alcohol use.

#### Sub-theme 2a: Social environmental facilitators of and barriers to alcohol use

Social and environmental factors present in society were seen to have a notable influence on alcohol use. Many participants reported an environmental context of alcohol being easily available to them, with several outlets selling alcohol, such as off licenses or pubs, nearby in the local area. Consequently, alcohol was considered to be highly accessible in the local neighbourhood. One participant expressed despondency about a contrast between the high availability of alcohol outlets and low availability of other outlets (e.g., a butchers) in their local area, and contrasted this with the higher availability of outlets for healthier products in more advantaged neighbourhoods. This contributed to a sense of low trust (see theme one) towards those implementing the placement of these outlets.

*“I just think it’s funny as well though*,* in all deprived areas there’s always a pub*,* always an off license*,* always a bookies … we should be thriving here*,* we should have butchers*,* fruit and veg*,* but do you know what I mean*,* they can always stick one of them [pub*,* off license*,* bookies] on the corner.” (FG5*,* Participant 1)*.

Places outside the house (e.g., a pub) to drink or buy alcohol were reported as common; *“I live right next door to a pub*,* so it’s only a 30 second walk” (FG4*,* Participant 5)*. However, availability was also discussed by some participants in terms of alcohol being present in the house as promoting drinking; *“I found if there’s a bottle of beer in the fridge*,* I say oh I’ll have it” (FG4*,* Participant 3)*. The accessibility of alcohol was suggested to promote alcohol use by making it an easy option, particularly in the context of limited other recreational activities. The environmental availability of alcohol was seen to promote alcohol use for some as a coping strategy for stressors in daily life in the absence of alternative resources or activities in the community (see sub-theme 2b).

*“It’s easy to get isn’t it … It’s just literally*,* if something happens here*,* you could walk down and get a bottle.” (FG6*,* Participant 1)*.

*“It’s [proximity] just encouraging it more isn’t it*,* like do you know what I mean*,* encouraging people to drink. It’s like*,* oh what shall we do*,* there’s nothing to do*,* let’s just go and have a drink*,* go and waste my money in the bookies.” (FG5*,* Participant 1)*.

A social acceptance of alcohol use, and social pressure to drink alcohol with others, was highlighted. Some participants discussed social norms of seeing alcohol use on television programmes, in particular on soap operas: *“every soaps round the pub” (FG2*,* Participant 3)*. However, one participant referred to the portrayal of drinking in a pub on soap operas as unrealistic due to the perceived unaffordability of alcohol. Moreover, limits to the social acceptance of alcohol were reported. Some participants suggested that other individuals would have negative attitudes towards instances of higher levels of alcohol use, and judgemental attitudes of waste collectors and neighbours in particular were referenced. Negative attitudes from others towards alcohol use were also suggested by some participants to have negative impacts on social relationships with family and friends.

*“I know for a fact the recycling people they do judge you.” (FG6*,* Participant 1)*.

Some participants highlighted the influence of their family as either a facilitator or barrier to their own alcohol use. They often brought up their own background in terms of their parents or other family members alcohol use, and discussed how this influenced their own alcohol use. Some participants referred to a family members moderate alcohol use as providing an example for themselves and other family members of how to consume alcohol. However, one participant highlighted that having a family member with higher rates of alcohol use made them less likely to drink alcohol; *“that’s another thing [observing family member with high alcohol use] like why I don’t like it” (FG5*,* Participant 1)*.

Work was highlighted as mainly preventing alcohol use due to the impacts on the following day. Most participants suggested that having to attend work the next day was seen to prevent alcohol use as it could negatively affect work performance, lead to a more difficult day, and could potentially lead to job loss if they failed to attend work.

*“We can’t drink every night cause we gotta go to work tomorrow … You don’t wanna start work at 5 o’clock and wake up at 9 o’clock [following alcohol use] like oh my god*,* I’m gonna lose my job.” (FG3*,* Participant 1)*.

Concurrently, not having to attend work the next day was seen to facilitate alcohol use for some participants; “*If you know you haven’t got work the next day*,* you don’t worry so much about it*,* cause like you know that you can take it a bit easier” (FG1).* Drinking alcohol after work was also reported; *“A lot of people drink every day when they come home from work” (FG2*,* Participant 2).*

Similarly, some participants suggested that lockdown during the COVID-19 pandemic was a positive experience and that they drank less alcohol during this time period.

*“I enjoyed lockdown … I really enjoyed the sun … I was around walking*,* walking to exercise a lot*,* I really enjoyed it. I didn’t buy alcohol at all.” (FG3*,* Participant 4)*.

In contrast, lockdowns were referred to as promoting alcohol use for some, for example, by drinking *“more times a week probably” (FG3*,* Participant 1)*. Moreover, one participant reported an increase in alcohol use following lockdown; *“It was when we had to come back out [from lockdown] and go to work and start doing things … So it was like getting back in to all that [work]*,* and then the pressure … we really started drinking then.” (FG3*,* Participant 2)*.

#### Sub-theme 2b: Motivations for and patterns of alcohol use

Accounts of alcohol use by participants tended to refer to their motivations for drinking alcohol, including the reasons for and intended outcomes of alcohol use. Accounts of alcohol use also included patterns of alcohol use, including behavioural reports of how alcohol is consumed. Alcohol use was seen as a *“coping strategy” (FG6*,* Participant 2)* across all focus groups, although discussion around drinking to forget about life issues and stresses was more common in focus groups two to six. Most participants expressed that they drank alcohol to distract from daily life and as an escape from an issue or problem in their daily lives; *“I would drink so I would forget” (FG6*,* Participant 1)*, with one suggesting that alcohol was used to *“try and detach from the day’s events” (FG4*,* Participant 3)*.

*“It’s your coping mechanism*,* it’s your go to*,* it’s your comfort blanket.” (FG2*,* Participant 3)*.

Some participants reported drinking in response to experiencing a bad day or week, such as due to social relationship issues. Participants emphasised that their alcohol use was dependent on what had happened during a day to affect their mood. Following a negative event occurring, alcohol was seen as easy to purchase due to its high availability in the local area (see sub-theme 2a). Drinking alcohol mainly at a weekend or Friday evening was also highlighted by participants.

*“If I had a bad day*,* … you’re just like ah fuck I’m having a drink*,* do you know what I mean*,* it is like a go to.” (FG6*,* Participant 1)*.

Most participants highlighted drinking alcohol to obtain effects of alcohol consumption such as increased relaxation and reduced stress and anxiety, such as to *“calm my nerves” (FG2*,* Participant 2)*. Stress was said to be related to pressure from daily life, work, bills, and interpersonal relationships (e.g., family).

*“I drink ‘cause it relaxes me.” (FG6*,* Participant 2)*.

Some participants suggested that these effects of alcohol are apparent whilst drinking alcohol or *“in the moment” (FG1)*. However, they said that these benefits only existed *“to a limit” (FG2*,* Participant 2)*, and were not maintained overtime; *“it’s not a quick fix” (FG6*,* Participant 1)*. Some participants suggested that alcohol use had negative mental health impacts in the long term, and expressed despondency that drinking alcohol did not address their reasons for alcohol use (e.g., daily life stress) by *“not getting rid of the problem*,* you’re just making it worse” (FG5*,* Participant 1)*.

*“I suffer really bad anxiety*,* so*,* and I know it [alcohol] makes it worse*,* but the first thing it does is make it better.” (FG2*,* Participant 3)*.

*“It [alcohol] can make you depressed.” (FG4*,* Participant 4)*.

All focus groups reported several negative health consequences of alcohol use, such as disease, organ damage (e.g., liver damage), alcohol poisoning, and premature death; *“organ failure” (FG5*,* Participant 1)*. Other reported negative consequences of alcohol use across focus groups two to six included financial impacts related to their bank balance and debt.

*“You’ll wake up the next day [following alcohol use] and think*,* god how much money did I spend.” (FG4*,* Participant 4)*.

Concurrently, a few participants reported perceived health benefits from alcohol use. These related to specific drinks, such as red wine, beer, and spirits (e.g., brandy, whiskey). Alcohol was reported to have some health benefits, including for sleep, some organs (e.g., kidneys, heart, and skin), improved blood circulation, and treating a cold.

“*There is a health benefit to alcohol of certain drinks.” (FG3*,* Participant 2)*.

*“It’s good for the heart.” (FG3*,* Participant 4)*.

A few participants across focus groups four to six emphasised that they drank alcohol as a habit due to boredom. They highlighted a lack of availability of alternative activities or resources other than alcohol; *“[There’s] nothing else to do” (FG5*,* Participant 1)*.

*“It’s a habit*,* yeah. Mines more of a routine*,* cause like*,* you’re bored.” (FG6*,* Participant 1)*.

However, drinking motives were considered to change over time for most participants. They reported that when they were younger, they drank alcohol for fun and out of the house (e.g., at a pub). Over time, this had changed to drinking alcohol to forget about or cope with life events. Life events promoting alcohol use over time included a change in housing status (e.g., losing a house) and loss of people (e.g., bereavement, loss of child custody).

*“When you’re younger you’re out [drinking alcohol] all the time.” (FG4*,* Participant 1)*.

Patterns of alcohol use were also discussed by participants. Some reported drinking a high quantity of alcohol at one time, or heavy episodic drinking; *“about a month ago*,* I had about 20 cans” (FG5*,* Participant 3)*. A preference for cheap high strength alcohol was emphasised by some participants.

*“We like to go round and buy the ones that are the cheapest.” (FG2*,* Participant 2)*.

*“I want the strongest thing I can get for the most cheapest thing that I can buy. That’s what I want.” (FG6*,* Participant 1)*.

A few participants discussed looking at the back of bottles for the alcohol strength as a deciding factor before purchase. A link between drinking alcohol and other health behaviours was referenced by one person; *“smoking and alcohol go hand in hand” (FG5*,* Participant 3)*. Most participants also reported mainly drinking alone at home, rather than out of the house; *“I don’t go out and drink*,* I drink in the house” (FG2*,* Participant 2)*.

All focus groups discussed a social component to their alcohol use. Attending social events and occasions (e.g., birthdays, Christmas) was seen to promote alcohol use. This was reported as a reason for not participating in Dry January by one participant; *“January is my birthday*,* so that’s another reason [for not participating in Dry January]” (FG3*,* Participant 2)*.

Risk perception of alcohol use for most participants was seen to be guided by an individual’s personal sense of risk rather than external guidance. It was perceived mainly in terms of the ability to drive a vehicle and risks of drink driving. Some participants highlighted that the impacts of alcohol use varied between individuals. This led them to use their own intuitive judgement when deciding how much alcohol to drink (*“it’s knowing your cut off point”; FG2*,* Participant 1)* rather than government set guidelines. Participants reported a need for personal control over their alcohol use; *“it’s good to have control” (FG3*,* Participant 3)*.

*“I know when I can and can’t drive*,* ‘cause of the amount of alcohol I drank. And nine times out of ten that’s not enough*,* so I drive. So it [unit guidelines] doesn’t make a difference to me.” (FG6*,* Participant 1)*.

## Discussion

This community-based qualitative study explored perspectives on alcohol public health interventions and experiences of alcohol use among a sample recruited through community organisations located in disadvantaged areas and workplaces of routine and manual occupations. A notable finding is that a lack of trust towards government alcohol intervention was salient in this sample and might represent a target to consider for alcohol interventions. We also identified targets including alcohol availability, coping with stress drinking motives, heavy episodic drinking, and cheap high strength alcohol consumption. The findings support explanations of the alcohol harm paradox focused on alcohol use in response to stress and as a coping strategy, drinking patterns (e.g., heavy episodic drinking), high density of places to buy alcohol, a lack of individual and neighbourhood resources, and family influence [3].

The lack of trust in government alcohol intervention suggested by this study’s findings could be linked to the wider literature of broader low trust in health interventions, for example, in relation to vaccines [78] and COVID-19 restrictions [79]. We suggest that this may align with the mediated trust model (developed from the health belief model [80]) which suggests that trust in government affects perceptions of the advantages and disadvantages of, and capacity to adhere to, government health advice [81]. Moreover, our findings may reflect that data collection was conducted following the COVID-19 pandemic, during which trust in government declined among disadvantaged populations [82]. However, it is not clear from the present study whether a lack of trust in government alcohol intervention may be common across SEP groups or differ by SEP. Whilst not explicitly stated among the sample in the present study, references to the government in terms of “them” and “they” may link to the rise of political populism and negative attitudes towards authorities, for example, in relation to Brexit. Political populism positions a population as in conflict with a perceived elite group [83]. Evidence suggests that populist views are negatively linked to the perception of public health interventions across health issues ranging from infection control to preventative healthcare [84]. Furthermore, our sample perceived that the government financially benefits from alcohol interventions, for example, the objectives of MUP tended to be misunderstood. However, there was less awareness that the alcohol industry financially benefits from sales and reduced restrictions [85]. Some negative views were also expressed among our sample towards the healthcare system. This may align with broader evidence of declining public satisfaction towards the NHS [86]. Suggestions to increase trust in health interventions include identifying trusted sources of interventions among different populations [87], collaborating with community organisations to deliver health interventions [88], and increasing public participation in policy development [89]. Alcohol interventions may need to increase trust towards the intervention source to increase acceptability among underserved populations. Future research could investigate which intervention sources for providing health advice about alcohol use would be most trusted by underserved populations.

The scepticism around the perceived effectiveness of alcohol interventions, such as anticipated outcomes of MUP, among our sample may align with the mediated trust model’s proposal that trust in government affects perceived disadvantages of adhering to government health advice [81]. Previous studies of objections to proposals to implement MUP include perceptions that it would: increase crime and other drug use, have negative economic impacts [90], and lead to individuals switching from drinking cheap strong cider to other drinks (e.g., vodka) [26]. However, evidence from MUP implementation indicates that concerns about unintended outcomes of the policy (e.g., increased crime) are largely inaccurate [91]. Moreover, the literature suggests that opposing arguments about MUP in public discourse tend to be propagated by the alcohol industry and economic think tanks [92]. Nevertheless, evaluation of MUP in Scotland indicates some reported negative financial impacts (e.g., prioritising alcohol purchase over other expenditures) among some low-income individuals [26] which should be acknowledged.

The motivations for and patterns of alcohol use identified in the present study may link to evidence in the wider literature that lower SEP populations drink alcohol to cope with stress [93] and demonstrated associations between alcohol use and poor mental health among lower SEP populations [94, 95]. Moreover, evidence has suggested that ill mental health (e.g., anxiety, depression) may explain an association between lower SEP and alcohol use [96]. One study indicated that the alcohol harm paradox may be explained by increased risks of aversive experiences, experiencing depression and anxiety, and coping drinking motives [97]. Whilst lower SEP populations are more likely to experience depression [98], they also experience reduced access to healthcare services [99]. The findings of the present study align with other qualitative studies which have found that lower SEP individuals drink alcohol to avoid thinking about negative daily life experiences [100] and to cope with stress [13, 14]. However, studies have found that higher SEP populations also report coping drinking motives for alcohol consumption [13, 100]. It may be possible that lower SEP populations drink alcohol to cope with different stressors than higher SEP populations, given that lower SEP populations experience higher rates of stressful life events [101]. Therefore, alcohol interventions could target coping drinking motives for stressors specific to experiences of lower SEP individuals.

The present study suggests that alcohol availability may play a role in promoting alcohol consumption, particularly among those in disadvantaged neighbourhoods. This supports evidence of an association between the density of alcohol outlets and an increased risk of alcohol use [102]. This may also align with availability theory which suggests that higher availability of alcohol is linked with increased alcohol use [103]. Evidence indicates that disadvantaged neighbourhoods have more alcohol outlets [104] and reducing the number of these outlets may reduce alcohol-related harms [105]. A review indicates that high availability of places to buy alcohol may promote a social norm of alcohol use in the absence of other neighbourhood recreational activities [106]. This supports the present study’s finding that the availability of alcohol in the local neighbourhood may promoting alcohol use in the context of a lack of alternatives. However, whilst our sample in the present study highlighted alcohol availability as a key factor promoting alcohol use, their recommendations for interventions tended to focus on individual lifestyle education. This may reflect a finding in the broader literature that public discourse on health promotion tends to focus on encouraging individuals to change their behaviours, rather than state intervention [107]. Within the present study, it may be noteworthy that there appeared to be a contradiction between low trust in the government for alcohol intervention and low trust towards those implementing the placement of alcohol outlets within disadvantaged neighbourhoods. Other identified potential social environmental influences suggested by our sample may align with the literature on family experiences as both a facilitator and barrier [17], employment as largely a barrier [108], social norms promoting alcohol use [109], and the impact of the COVID-19 pandemic in promoting alcohol use at home [110].

### Implications for interventions

Qualitative exploration of a phenomenon and attitudes towards interventions is an important component of the early stages of the person-based approach to intervention development. A practical outcome of this stage is the identification of factors which can be targeted within interventions [44]. The following implications for interventions should considered in the context that not all focus groups were presented with all possible interventions in the topic guide, as decisions were made on the basis of what was necessary to prompt discussion or allow existing discussion to continue. Our sample also had varied levels of alcohol consumption. Nevertheless, the present study’s findings indicate that alcohol interventions targeted towards the present sample could address the following targets related to a lack of trust towards government alcohol intervention. These include a lack of trust towards the motivations of government alcohol intervention (e.g., perception of a lack of government care about alcohol-related health harms) and scepticism about the perceived effectiveness of government alcohol intervention (e.g., perception of negative outcomes). Interventions could also address social environmental influences (e.g., alcohol availability) and motivations for and patterns of alcohol use (e.g., coping drinking motives for daily life stressors and drinking patterns including heavy episodic drinking and consumption of cheap high strength alcohol).

These intervention targets have implications for alcohol public health interventions based on the UK Government’s Alcohol Strategy of 2012 [19]. MUP may need to address the specific reasons reported for alcohol consumption among underserved populations to increase its acceptability. Similarly, the public health campaign Dry January may need to account for underlying motivations for alcohol consumption in its promotion of abstinence from alcohol during January. Moreover, the public health campaign Drink Free Days could consider how drinking patterns may affect alcohol-related harm within its promotion of taking more days per week on which alcohol is not consumed. For example, an individual could both be not consuming alcohol on several days per week, but at the same time engaging in heavy episodic drinking on other days of the week. Whilst alcohol abstinence is associated with health benefits [42], there are health risks associated with heavy episodic drinking [111]. The unit consumption guidance in the UK could account for perceived limited usability by considering different communication modes, such as the suggestion among our sample to use visual methods. In addition, high availability of alcohol outlets in underserved neighbourhoods and consumption of cheap high strength alcohol are likely best addressed by implementing policies including restrictions on alcohol availability and MUP, as recommended by the World Health Organization [18]. Overall, trust towards alcohol interventions among lower SEP populations may be increased by improved communication of the purpose, motivations, and intended outcomes of interventions. For example, greater rationale for specific alcohol interventions implemented by governments could be communicated to the general public.

### Strengths and limitations

This study has strengths in applying a novel health psychology approach to exploring alcohol intervention perspectives and consumption among our sample, incorporating a psychosocial approach [4]. This study extends the literature by incorporating a focus on identifying targets for future interventions and adapting the implementation of existing interventions, guided by the person-based approach to intervention development [44]. This study highlights the value of in-person recruitment and data collection in community-based settings, aligning with evidence on the benefits of community-based qualitative research for exploring health inequalities in underserved communities [112, 113]. This may be particularly pertinent for investigation of the alcohol harm paradox, for which research tends to typically employ quantitative approaches. Moreover, recruitment rates in the present study indicate that community organisation-based strategies were more effective than workplace-based strategies for recruitment to focus groups. This may be because the relatively substantial time commitment required to participate in a focus group may not align with the needs of a workplace. Future research may wish to build upon our recruitment strategy for further investigation of health inequalities.

However, the sample was not homogenous and some participants reflected diverse individual socioeconomic indicators (e.g., higher IMD scores), which may limit transferability of findings. However, these participants tended to also have lower indicators of income and education, and routine and manual occupations; which are other indicators of lower SEP. More substantial involvement of community-led recruitment and data collection may have led to further recruitment of less advantaged individuals. For example, training members of the public to recruit and conduct research in their own communities has been suggested as one strategy [54, 114, 115], although this would require further investment in terms of time and funding. Moreover, whilst our recruitment strategy was informed by relevant literature, it may have excluded individuals not involved in community-based organisations or in employment, who may in fact reflect the most disadvantaged individuals [116]. Nevertheless, this exploratory study took an inclusive and pragmatic recruitment approach to explore the feasibility of different strategies for recruiting underserved populations. Socioeconomic disadvantage is suggested to extend beyond solely single indicators (e.g., income), and reflect various other aspects of underserved communities related to occupation, education, social class, and access to services. It is therefore not surprising that our sample was not homogenous, as this reflects community recruitment settings in which individuals are likely to be comprised of complex social factors. Recommendations on recruiting underserved communities highlight the need to consider SEP holistically, rather than just focusing on one indicator (e.g., finances) [54].

## Conclusions

This community-based qualitative study indicates that lack of trust in government motivations for alcohol interventions and scepticism about perceived intervention effectiveness were salient in this sample and may represent important considerations for alcohol intervention design. Future research should investigate levels of trust towards the government for providing health advice about alcohol use, and how trust may be affected by SEP. Future research could also examine which sources of alcohol interventions would be preferred and acceptable to different SEP populations. Further intervention targets may include the high availability of alcohol in the local environment, as well as specific motivations for and patterns of alcohol use. Interventions which are tailored to the factors identified in this study may have increased acceptability among disadvantaged individuals.

## Supplementary Information

Supplementary Material 1.

## Data Availability

Data for the current study are not available. This is to maintain confidentiality of participant data.

## Abbreviations

SEP: Socioeconomic position
UK: United Kingdom
CHICO: CHecklist for Inclusive COmmunity involvement in health research
MUP: Minimum unit price
IMD: Index of Multiple Deprivation
NHS: National Health Service
