# Supplementary material for: A community-based qualitative study of perspectives on alcohol public health interventions and experiences of alcohol use
Source: BMC Public Health. 2026 Jul 17;26:2939. doi: 10.1186/s12889-026-28513-8 (PMC13656483; doi:10.1186/s12889-026-28513-8)
Supplement: Supplementary file 1 [file 12889_2026_28513_MOESM1_ESM.docx]

**Supplementary Materials**

**Appendix A. Standards for Reporting Qualitative Research (SRQR).**

|  | **Standards for Reporting Qualitative Research (SRQR)*** |  |
| --- | --- | --- |
|  | <http://www.equator-network.org/reporting-guidelines/srqr/> |  |
|  |  | **Page/line no(s).** |
| **Title and abstract** | |  |
|  | **Title** - Concise description of the nature and topic of the study Identifying the study as qualitative or indicating the approach (e.g., ethnography, grounded theory) or data collection methods (e.g., interview, focus group) is recommended | 1 |
|  | **Abstract** - Summary of key elements of the study using the abstract format of the intended publication; typically includes background, purpose, methods, results, and conclusions | 2-3 |
|  |  |  |
| **Introduction** | |  |
|  | **Problem formulation** - Description and significance of the problem/phenomenon studied; review of relevant theory and empirical work; problem statement | 4-9 |
|  | **Purpose or research questio**n - Purpose of the study and specific objectives or questions | 9 |
|  |  |  |
| **Methods** | |  |
|  | **Qualitative approach and research paradigm** - Qualitative approach (e.g., ethnography, grounded theory, case study, phenomenology, narrative research) and guiding theory if appropriate; identifying the research paradigm (e.g., postpositivist, constructivist/ interpretivist) is also recommended; rationale** | 9, 15-17 |
|  | **Researcher characteristics and reflexivity** - Researchers’ characteristics that may influence the research, including personal attributes, qualifications/experience, relationship with participants, assumptions, and/or presuppositions; potential or actual interaction between researchers’ characteristics and the research questions, approach, methods, results, and/or transferability | 17 |
|  | **Context** - Setting/site and salient contextual factors; rationale** | 9-11 |
|  | **Sampling strategy** - How and why research participants, documents, or events were selected; criteria for deciding when no further sampling was necessary (e.g., sampling saturation); rationale** | 14-15 |
|  | **Ethical issues pertaining to human subjects** - Documentation of approval by an appropriate ethics review board and participant consent, or explanation for lack thereof; other confidentiality and data security issues | 9 |
|  | **Data collection methods** - Types of data collected; details of data collection procedures including (as appropriate) start and stop dates of data collection and analysis, iterative process, triangulation of sources/methods, and modification of procedures in response to evolving study findings; rationale** | 11-14 |
|  | **Data collection instruments and technologies** - Description of instruments (e.g., interview guides, questionnaires) and devices (e.g., audio recorders) used for data collection; if/how the instrument(s) changed over the course of the study | 11-14 |
|  | **Units of study** - Number and relevant characteristics of participants, documents, or events included in the study; level of participation (could be reported in results) | 14-15 |
|  | **Data processing** - Methods for processing data prior to and during analysis, including transcription, data entry, data management and security, verification of data integrity, data coding, and anonymization/de-identification of excerpts | 16 |
|  | **Data analysis** - Process by which inferences, themes, etc., were identified and developed, including the researchers involved in data analysis; usually references a specific paradigm or approach; rationale** | 15-17 |
|  | **Techniques to enhance trustworthiness** - Techniques to enhance trustworthiness and credibility of data analysis (e.g., member checking, audit trail, triangulation); rationale** | 15-17 |
|  |  |  |
| **Results/findings** | |  |
|  | **Synthesis and interpretation** - Main findings (e.g., interpretations, inferences, and themes); might include development of a theory or model, or integration with prior research or theory | 17-31 |
|  | **Links to empirical data** - Evidence (e.g., quotes, field notes, text excerpts, photographs) to substantiate analytic findings | 17-31 |
|  |  |  |
| **Discussion** | |  |
|  | **Integration with prior work, implications, transferability, and contribution(s) to the field -** Short summary of main findings; explanation of how findings and conclusions connect to, support, elaborate on, or challenge conclusions of earlier scholarship; discussion of scope of application/generalizability; identification of unique contribution(s) to scholarship in a discipline or field | 31-36 |
|  | **Limitations** - Trustworthiness and limitations of findings | 36-38 |
|  |  |  |
| **Other** | |  |
|  | **Conflicts of interest** - Potential sources of influence or perceived influence on study conduct and conclusions; how these were managed | 39 |
|  | **Funding** - Sources of funding and other support; role of funders in data collection, interpretation, and reporting | 39 |
|  |  |  |
|  | *The authors created the SRQR by searching the literature to identify guidelines, reporting standards, and critical appraisal criteria for qualitative research; reviewing the reference lists of retrieved sources; and contacting experts to gain feedback. The SRQR aims to improve the transparency of all aspects of qualitative research by providing clear standards for reporting qualitative research. |  |
|  |  |  |
|  | **The rationale should briefly discuss the justification for choosing that theory, approach, method, or technique rather than other options available, the assumptions and limitations implicit in those choices, and how those choices influence study conclusions and transferability. As appropriate, the rationale for several items might be discussed together. |  |
|  |  |  |
|  | **Reference:** |  |
|  | O'Brien BC, Harris IB, Beckman TJ, Reed DA, Cook DA. **Standards for reporting qualitative research: a synthesis of recommendations.** *Academic Medicine*, Vol. 89, No. 9 / Sept 2014  DOI: 10.1097/ACM.0000000000000388 |  |
|  |  |  |
|  |  |  |

**Appendix B. Focus group topic guide.**

**Part 1 – Questions on interventions**

**We are interested in finding out what you think about health initiatives and advice that help people to cut down on their drinking. Can anyone think of anything they have seen like this?**

- **These are the government recommended guidelines (show image).**
- What do you think about them? Do you use them?
- Do you think they’re useful for you? Why? Would they be useful for anyone in particular?
- Who do you think they’re aimed at?
- Who do you think would find them most useful?
- **Scotland has a minimum unit price policy, which is X (show image).**
- What do you think about it? Would you like it here?
- Do you think it’s useful for you? Why? Would it be useful for anyone in particular?
- Who do you think it’s aimed at?
- Who do you think would find it most useful?

One of the following:

- **This is a government supported campaign called Dry January (show image).**
- What do you think about it? Have you used it?
- Do you think it’s useful for you? Why? Would it be useful to anyone in particular?
- Who do you think it’s aimed?
- Who do you think would find it most useful?

Or

- **This is a government supported campaign called Drink Free Days (show image).**
- What do you think about it? Have you used it?
- Do you think it’s useful for you? Why? Would it be useful for anyone in particular?
- Who do you think it’s aimed?
- Who do you think would find it most useful?
- **In England, pub open hours are generally 11am – 11pm, bar/club open hours can be until 2am – 5am, and some places like supermarkets may have 24 hours licenses.**
- Do you think that licencing hours are a good or bad thing?

**Part 2 – General introduction**

- **Thinking back to the last time you had a drink…**
- What day of the week was it?
- Where were you? Who were you with?
- What were you drinking? How much were you drinking?
- Where did you get it? E.g., shop, pub/club.

**Part 3 – Questions on consumption**

- **Why do you think people drink alcohol?**
- What are some of the things that might influence whether people drink?
- **Do you think there are any positive aspects of drinking alcohol?** If so, what aspects?
- Are there any negative aspects of drinking alcohol? If so, what aspects?
- **Do you tend to drink at a particular place? If so, where?**
- Do you tend to drink alone or with others? If others, who?
- Do you tend to drink at a particular time? If so, when?
- Does your drinking changes over the week? If so, how?
- Do you think your drinking has changed over your life? If so, how?
- **Do you think your drinking is affected by daily life? If so, how?**
- Do you think you drink for any particular reasons?
- Do you think lockdowns and the pandemic affected your drinking? If so, how?
- How does alcohol make you feel?
- Do you think your drinking is linked to wellbeing?
- **Do you think your drinking is affected by the price of alcohol?**
- Does price affect what you drink? Does price affect how much you drink? Does price affect where you drink?
- Do you think your drinking is affected by adverts or promotions for alcohol?

**Appendix C. AUDIT-C analyses.**

**Figure S1.** AUDIT-C question 1 answers across 23 participants.

**Figure S2.** AUDIT-C question 2 answers across 21 participants.

*Note.* FG5 P1 and FG5 P2 did not complete this question.

**Figure S3.** AUDIT-C question 3 answers across 22 participants.

Note. FG5 P4 did not complete this question.
